# Editorial

**Published:** 1842-09-03

**Authors:** 


### THE MEDICAL EXAMINER.

### PHILADELPHIA, SEPTEMBER 3, 1842.

In a late number of the Bulletin of the Royal Academy of Medicine we observe a notice of the death of Dr. Double, one of the most distinguished of its members. Dr. Double is the author of a large work on Semeiology, published some years since, and of numerous essays and reports made to the Academy of Medicine: but of late years an extended practice drew so heavily upon his time, as to leave him little leisure for the literary labours of the profession. He was, however, so strongly attached by his classical taste to the study of the older and now almost neglected medical writers, that he took a decided part in the discussion which followed the reading of a capital memoir upon the writings of Galen, by M. Dubois, (of Amiens.) Confessing our own sin of comparative neglect of the classical medical authorities, we are not of the number of those who regard the study of the ancients as a futile, because unnecessary pursuit. The tendency of medicine is now practical, eminently so; but are we not wandering away too far from the earlier cultivators of our science? If their facts are now of little value, because we know so much more, we lose the original modes of thought, the earnest reflection which form a good counterpoise to the exactness, but at the same time hardness of our modern authors.

Dr. Pariset, in his eulogium, hints that Dr. Double, like many other physicians, was but an indifferent practitioner in his own case, temporising and trusting to his own biassed judgment, until decided treatment came too late.

---

At the recent elections in France, the following medical men were elected members of the Chamber of Deputies:—Dr. Bouillaud, a clinical professor of medicine in the Faculty of Medicine at Paris, one of the physicians of La Charité, and celebrated as the author of an excellent treatise on the Diseases of the Heart; M. Terne, the Mayor of Lyons; and M. Dezeimeris, the Librarian of the Faculty of Medicine, and the well known bibliographist.

---

M. Auguste Berard, Surgeon to the Neckar Hospital, has been appointed to the Chair of Clinical Surgery in the Faculty of Paris, vacant by the death of M. Sansom. This *concours* was celebrated for its brilliancy, and the result will, we doubt not, meet with general approbation. M. BERARD is the brother of the present Professor of Physiology, and his general superiority as a surgeon is incontestible.

Dr. LIEBIG, whose recent investigations and publications in Organic Chemistry have become so justly celebrated, has been elected a corresponding member of the French Institute, in the section of Chemistry of the Academy of Sciences.

